# Treatment of inflammatory arthritis via targeting of tristetraprolin, a master regulator of pro-inflammatory gene expression

**DOI:** 10.1136/annrheumdis-2016-209424

**Published:** 2017-02-17

**Authors:** E A Ross, A J Naylor, J D O'Neil, T Crowley, M L Ridley, J Crowe, T Smallie, T J Tang, J D Turner, L V Norling, S Dominguez, H Perlman, N M Verrills, G Kollias, M P Vitek, A Filer, C D Buckley, J L Dean, A R Clark

**Affiliations:** 1 Institute of Cardiovascular and Medical Sciences, University of Glasgow, Glasgow, UK; 2 Institute of Inflammation and Ageing, University of Birmingham, Birmingham, UK; 3 Kennedy Institute of Rheumatology, University of Oxford, Oxford, UK; 4 William Harvey Research Institute, QMUL, London, UK; 5 Division of Rheumatology, Northwestern University, Chicago, Illinois, USA; 6 School of Biomedical Sciences and Pharmacy, University of Newcastle, Callaghan, New South Wales, Australia; 7 Division of Immunology, Biomedical Sciences Research Center ‘Alexander Fleming’, Vari, Greece; 8 Cognosci Inc., Research Triangle Park, North Carolina, USA

**Keywords:** Fibroblasts, Inflammation, Rheumatoid Arthritis, TNF-alpha, Cytokines

## Abstract

**Objectives:**

Tristetraprolin (TTP), a negative regulator of many pro-inflammatory genes, is strongly expressed in rheumatoid synovial cells. The mitogen-activated protein kinase (MAPK) p38 pathway mediates the inactivation of TTP via phosphorylation of two serine residues. We wished to test the hypothesis that these phosphorylations contribute to the development of inflammatory arthritis, and that, conversely, joint inflammation may be inhibited by promoting the dephosphorylation and activation of TTP.

**Methods:**

The expression of TTP and its relationship with MAPK p38 activity were examined in non-inflamed and rheumatoid arthritis (RA) synovial tissue. Experimental arthritis was induced in a genetically modified mouse strain, in which endogenous TTP cannot be phosphorylated and inactivated. In vitro and in vivo experiments were performed to test anti-inflammatory effects of compounds that activate the protein phosphatase 2A (PP2A) and promote dephosphorylation of TTP.

**Results:**

TTP expression was significantly higher in RA than non-inflamed synovium, detected in macrophages, vascular endothelial cells and some fibroblasts and co-localised with MAPK p38 activation. Substitution of TTP phosphorylation sites conferred dramatic protection against inflammatory arthritis in mice. Two distinct PP2A agonists also reduced inflammation and prevented bone erosion. In vitro anti-inflammatory effects of PP2A agonism were mediated by TTP activation.

**Conclusions:**

The phosphorylation state of TTP is a critical determinant of inflammatory responses, and a tractable target for novel anti-inflammatory treatments.

## Introduction

The mitogen-activated protein kinase (MAPK) p38 signalling pathway positively regulates the expression of many inflammatory mediators, and is thought to play a key role in the pathogenesis of rheumatoid arthritis (RA).^1–3^ Although activation of all four MAPK p38 isoforms (α, β, γ and δ) has been detected in RA synovial cells,^3^ genetic and pharmacological evidence suggests that MAPK p38α is likely to be the most important contributor to joint inflammation and bone erosion.^1^ ^2^ Prototypical inhibitors of MAPK p38α and β were discovered in a high-throughput screen for compounds that reduced the expression of tumour necrosis factor α (TNFα) in activated macrophages.^4^ Increasingly specific and potent inhibitors were later generated by several pharmaceutical companies. These compounds reduced the expression of many inflammatory mediators and they also demonstrated therapeutic effects in experimental models of RA. Therefore, they were considered promising as disease-modifying antirheumatic drugs,^2^ ^5^ and several entered clinical trials in RA. Yet no MAPK p38 inhibitor has received regulatory approval, seemingly due to dose-limiting hepatotoxicity and lack of sustained anti-inflammatory efficacy.^6^ The following questions arise: Why, contrary to expectation, did inhibition of MAPK p38 fail to achieve lasting therapeutic effects? How does the MAPK p38 pathway regulate inflammatory responses? Can our understanding of the pathway still be leveraged to devise more effective anti-inflammatory strategies?

Many of the effects of MAPK p38 on the expression of pro-inflammatory genes are mediated by the mRNA-binding protein tristetraprolin (TTP). TTP binds to adenosine/uridine-rich elements (AREs) in the 3′ untranslated regions of target mRNAs. It then recruits deadenylases, which shorten the poly(A) tail, resulting in suppression of translation and rapid degradation of the mRNA.^7^ ^8^ Disruption of the mouse TTP gene (formally *Zfp36*) results in a severe inflammatory syndrome, including an erosive arthritis that resembles RA.^8^ ^9^ Dysregulated expression of TNFα by macrophages is necessary but not sufficient for the RA-like phenotype.^10^ ^11^ Either disruption of the *Zfp36* gene or targeted deletion of the *Tnf* ARE renders TNF biosynthesis insensitive to MAPK p38 inhibition.^12^ ^13^ This indicates that MAPK p38 enhances TNF expression by inactivating the anti-inflammatory protein TTP.

Several groups have characterised the mechanism of regulation of TTP expression and function.^14–20^ MAPK p38 is activated in response to various pro-inflammatory stimuli, and in turn activates the downstream kinase MAPK-activated protein kinase 2 (MK2). MK2 phosphorylates serines 52 and 178 of murine TTP (60 and 186 of human TTP).^21^ ^22^ These phosphorylations have seemingly contradictory effects on the expression and activity of TTP. First, they protect it from destruction by the proteasome.^17^ ^18^ ^23^ Second, they inactivate it by impairing its ability to recruit deadenylases,^16^ ^24^ ^25^ reducing its affinity for RNA^26^ or both. We recently described a knockin mouse strain (*Zfp36aa/aa*), in which serine 52 and 178 codons of the endogenous *Zfp36* locus were substituted by alanine codons. These mice express a mutant form of TTP (known as TTP-aa), which cannot be phosphorylated by MK2. It was expressed at low levels, yet functioned as a potent suppressor of many inflammatory mediators.^15^ Furthermore, the inflammatory consequences of dysregulated MAPK p38 signalling were mitigated by substitution of the two TTP phosphorylation sites.^20^

A result of the coupled stabilisation and inactivation of TTP is that MAPK p38 activation promotes the accumulation of TTP in its inactive form. As the activity of the MAPK p38 pathway declines, TTP is dephosphorylated by protein phosphatase 2A (PP2A),^27^ converting it to an active form that promotes degradation of target mRNAs. Because of this complex regulation of expression and activity of TTP, acute and chronic inhibition of MAPK p38 have very different effects on inflammatory mRNA stability.^28^ Addition of a MAPK p38 inhibitor after a pro-inflammatory stimulus causes activation of pre-existing TTP and enhanced degradation of target mRNAs. In contrast, prolonged inhibition of MAPK p38 prevents the accumulation of TTP protein, and target mRNAs consequently remain stable. This phenomenon may contribute to the transient anti-inflammatory effects of MAPK p38 inhibitors in clinical trials.^1^ ^29^ Additional mechanisms of escape from anti-inflammatory effects of the inhibitors have also been suggested,^1^ ^29^ mostly involving the disruption of MAPK p38-dependent negative feedback loops.

Aberrant activation of MAPK p38 signalling in the RA synovium has been described.^3^ TTP protein has been detected in the RA synovium,^30^ but the cells expressing it were not identified, and its relationship with disease state or with MAPK p38 activity was not explored. Here, we wished to investigate whether inactivation of TTP via the phosphorylation of serines 52 and 178 contributes to synovial inflammation and bone erosion, and whether those pathological processes might be prevented by re-activation of TTP.

## Methods

Detailed methods are provided in the online supplementary material.

10.1136/annrheumdis-2016-209424.supp1 Supplementary material

### Human tissue

Synovial tissue biopsies were obtained from patients undergoing ultrasound-guided arthroscopy. Samples were designated ‘normal’ if histological inspection revealed no macroscopic evidence of inflammation, and absence of inflammatory pathology was confirmed by clinical follow-up. All participants gave written informed consent. The study was approved by the National Research Ethics Service Committee West Midlands.

### Animal studies

All mice were maintained and housed under conventional conditions in the Biomedical Services Unit at the University of Birmingham. Experimental protocols were performed under Home Office guidelines and project licence 40/8003. *Zfp36aa/aa*^15^ and TNFΔARE^12^ strains have been previously described.

### Statistical analysis

Clinical scoring, histological analyses and scoring of bone erosion were performed by ‘blinded’ observers. Statistical analysis was performed by Mann-Whitney U test using Prism V.6.0 (GraphPad).

## Results

### Expression of TTP in the rheumatoid synovium

**Figure 1:**
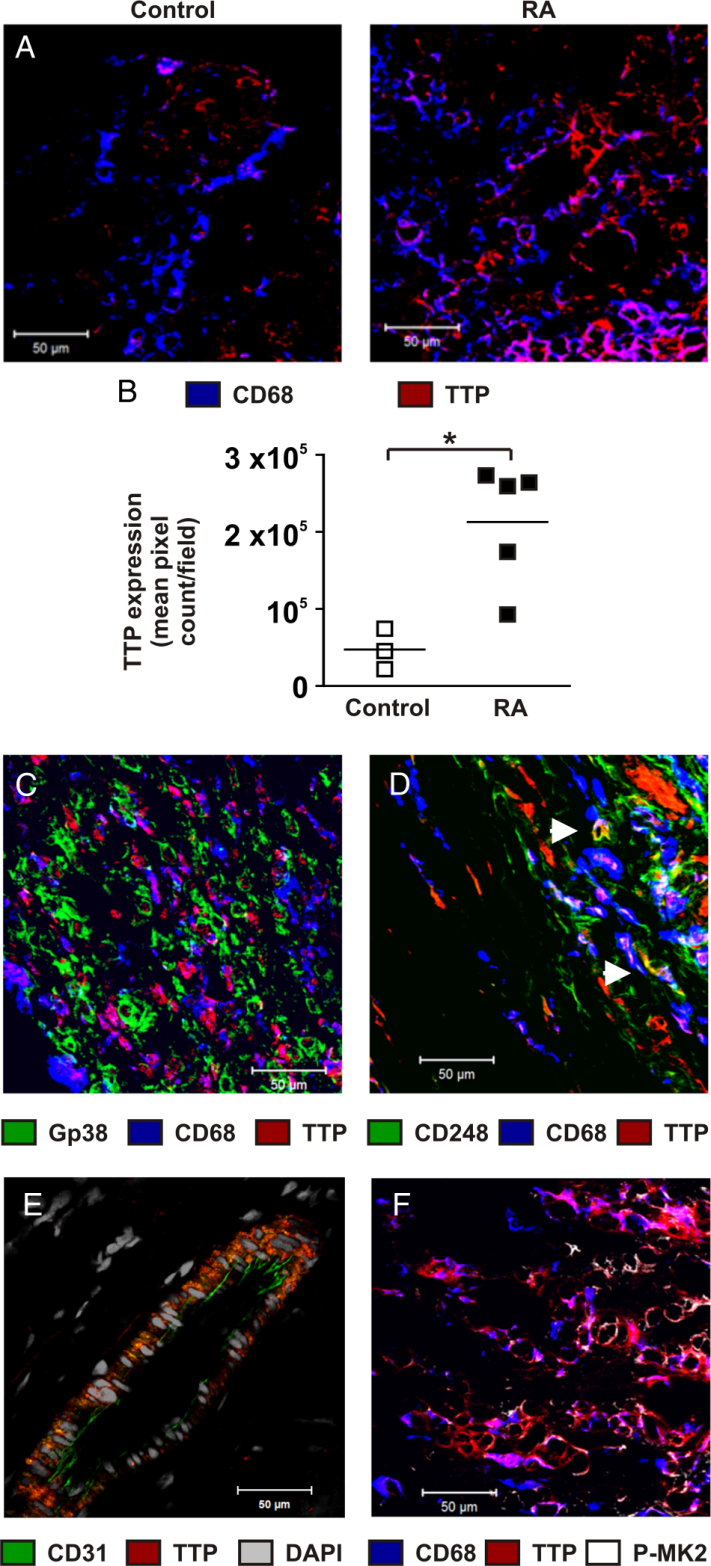
Expression of tristetraprolin (TTP) in the rheumatoid synovium. Biopsies from non-inflamed (A, left) or rheumatoid arthritis (RA) synovium (A, right, C-F) were stained using antibodies with the specificities indicated, and with DAPI (4'6-diamidino-2'-phenylindole) nuclear stain. The nuclear stain is shown only in (D), to identify the topology of the vessel wall. Antibody isotype-matched control stains were essentially blank (see online supplementary figure S1). (B) Sections of non-inflamed or RA synovial tissue were stained with antibody against TTP, and staining was quantified using ImageJ, by pixel counting of three fields per section, selected and scanned in blinded manner. *p<0.05 (Mann-Whitney U test).

Expression of TTP protein was significantly elevated in synovial tissue of patients with RA compared with non-inflamed controls (figure 1A, B). TTP was present in several cell types, with strongest staining in CD68+ macrophages. The glycoproteins podoplanin (PDPN) and CD248 are thought to identify discrete populations of fibroblasts in the rheumatoid synovium.^31^ There was little co-localisation of PDPN with TTP. Instead, PDPN+ fibroblasts were interspersed with TTP+ CD68+ macrophages (figure 1C). A subset of CD248+ cells expressed TTP (figure 1D). There was also strong staining for TTP in CD31+ vascular endothelial cells (figure 1E, see online supplementary figure S2). TTP protein was co-localised with activated MK2 in the cytoplasm of many macrophages (figure 1F). Antibody controls and single colour images are in online supplementary figures S1 and S2.

### Blockade of TTP phosphorylation is protective in experimental models of inflammation and inflammatory arthritis

**Figure 2:**
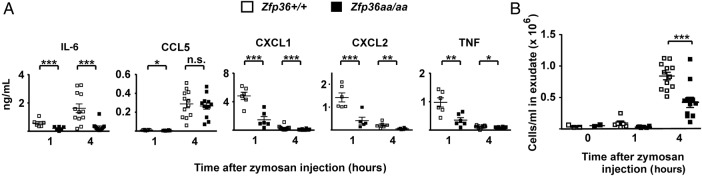
*Zfp36aa/aa* mice are protected from zymosan-induced local inflammation. Dorsal air pouches were created and localised inflammation was induced by injection of 7 μL of 1 mg/mL zymosan. After 1 or 4 hours mice were humanely culled. (A) Cytokines and chemokines were measured in air pouch exudate fluid. Graphs represent means±SEM from 6 mice (1 hour) or 12 mice (4 hours) of each genotype. Basal expression of all cytokines and chemokines was negligible in untreated mice (data not shown). (B) Cells in exudate fluid were counted using a haemocytometer. n.s., not statistically significant; *p<0.05; **p<0.01; ***p<0.005 (Mann-Whitney U test).

To test the hypothesis that phosphorylation of TTP favours joint inflammation, we employed *Zfp36aa/aa* mice.^15^ Initially, responses were tested in the zymosan-induced air pouch inflammation model, which recapitulates aspects of the localised synovial inflammation observed in RA.^32^ Injection of zymosan into air pouches of *Zfp36+/+* mice rapidly elevated levels of CXCL1 (chemokine (C-X-C motif) ligand 1), CXCL2, interleukin (IL)-6 and TNF in the exudate fluid. These cytokines are known to be regulated by TTP,^8^ ^15^ and were all underexpressed in exudate fluid of *Zfp36aa/aa* mice at 1 and 4 hours time points (figure 2A). In contrast, CCL5 was strongly elevated at 4 hours but not significantly different between *Zfp36+/+* and *Zfp36aa/aa* mice. *Ccl5* mRNA does not possess an ARE and has not been implicated as a TTP target. This result indicates that cytokine responses are selectively rather than globally affected by the TTP mutation. Zymosan also promoted an influx of leucocytes (figure 2B), the majority of which were neutrophils (data not shown). Cellular infiltration was decreased by 50% in *Zfp36aa/aa* mice.

**Figure 3:**
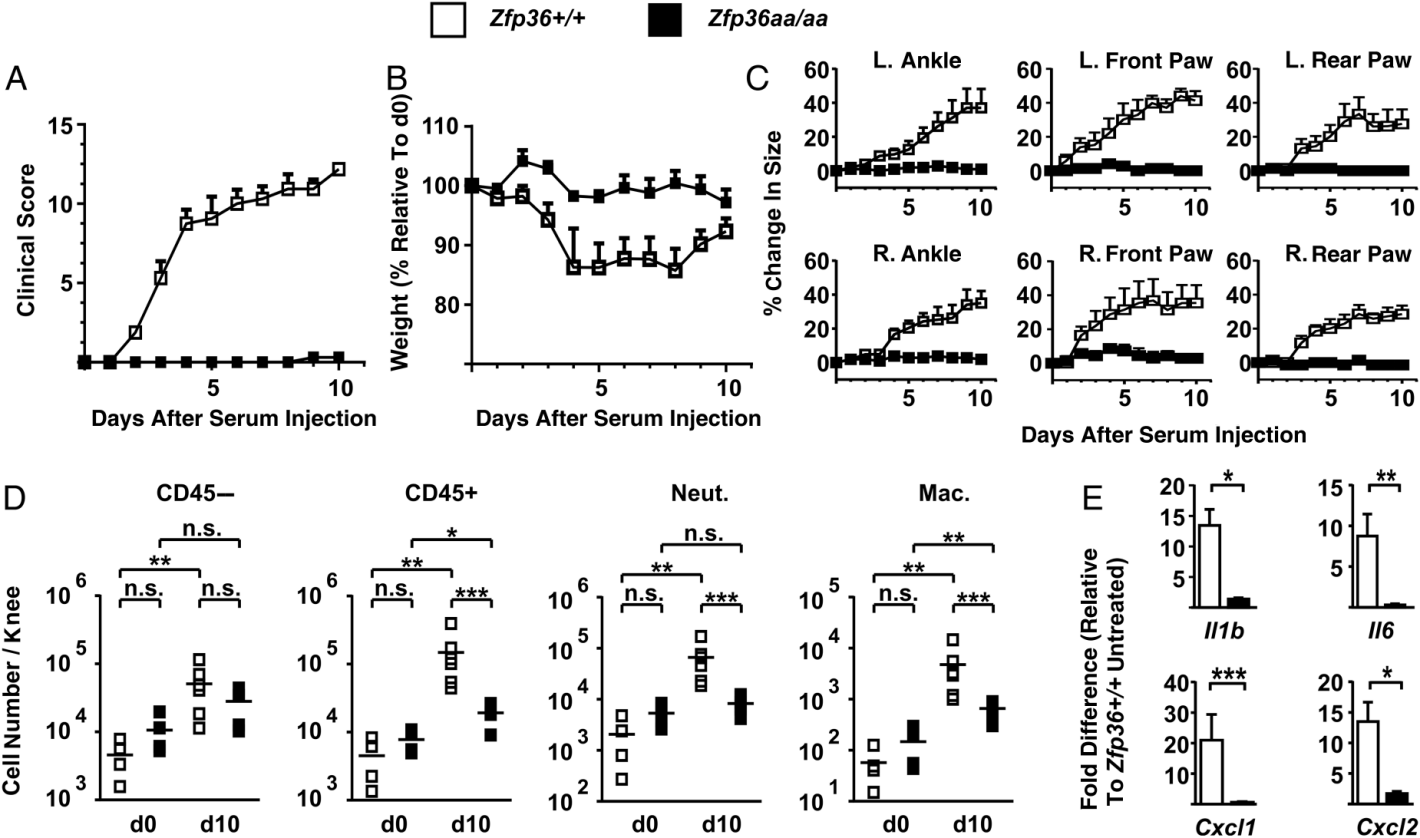
*Zfp36aa/aa* mice are resistant to experimental arthritis. *Zfp36+/+* and *Zfp36aa/aa* mice were injected intraperitoneal with 75 μL of arthritogenic serum from K/BxN donor mice on days 0 and 1. (A) Clinical score and (B) weight were monitored over 10 days. (C) Ankle and foot pad thickness were measured. Graphs represent mean±SEM (n=6). (D) Feet were digested at d0 or d10, CD45− and CD45+ cells, neutrophils and macrophages were counted using flow cytometry. Graphs represent mean±SEM (n=6). n.s., not statistically significant; *p<0.05; **p<0.01; ***p<0.005 (Mann-Whitney U test). (E) At d10, RNA was recovered from digested wrist joints of three mice of each genotype, and selected cytokine and chemokine mRNAs were measured by quantitative PCR, with normalisation first against *Gapdh* then against paws of vehicle-treated, uninflamed *Zfp36+/+* mice. Graphs represent mean±SEM (n=3). *p<0.05; **p<0.01; ***p<0.005 (Mann-Whitney U test).

Serum transfer-induced arthritis (STIA)^33^ was attempted next. All *Zfp36+/+* mice developed arthritis after injection of arthritogenic K/BxN serum (figure 3A), with transient weight loss (figure 3B) and swelling of ankles and footpads (figure 3C). STIA was associated with increased numbers of CD45− cells in knee joints due to stromal cell proliferation (figure 3D); marked infiltration of CD45+ cells, the majority of which were neutrophils and macrophages (figure 3D) and elevated expression of the pro-inflammatory mRNAs *Il1b*, *Il6*, *Cxcl1* and *Cxcl2* (figure 3E). These changes were absent or almost absent from serum-injected *Zfp36aa/aa* mice.

**Figure 4:**
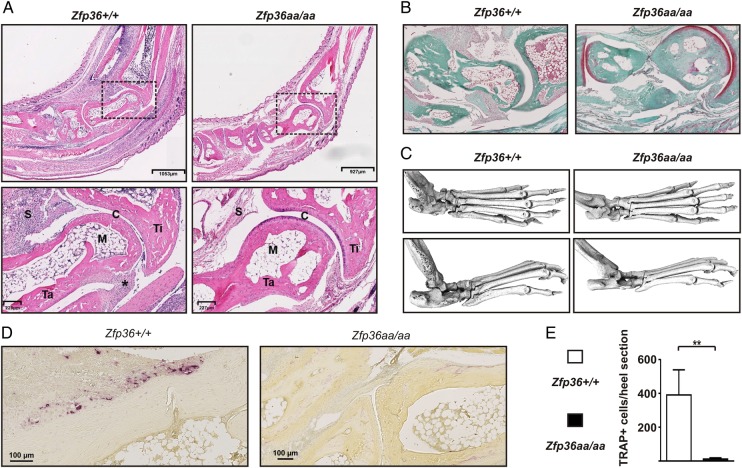
*Zfp36aa/aa* mice are resistant to experimental arthritis. Experimental arthritis was induced as in figure 3. (A) Representative H&E stained sagittal sections of *Zfp36+/+* and *Zfp36aa/aa* heel regions at d10. The inset boxes in upper panels are shown at higher resolution in lower panels. C, cartilage; S, synovium; M, bone marrow; Ta, talus; Ti, tibia; *, enthesitis. (B) Similar sections were stained with fast green and Safranin O to identify articular cartilage (red stain). Representative images are shown. (C) Whole feet were subjected to μCT. Representative images are shown from top and side. (D) Activated osteoclasts were visualised by staining for tartrate-resistant acid phosphatase (TRAP; dark red stain) and (E) TRAP-positive cells per heel section were counted. The graph represents mean±SEM (n=6). **p<0.01 (Mann-Whitney U test).

In *Zfp36+/+* mice, K/BxN serum induced leucocytic infiltration of joints and skin, synovial hyperplasia and pannus formation (figure 4A), loss of articular cartilage (figure 4A, B) and peri-articular erosion of bone (figure 4A, C), which was accompanied by osteoclast activation (figure 4D, E). None of these changes occurred in *Zfp36aa/aa* mice. Similar experiments have been carried out four times, using three batches of K/BxN serum from two laboratories. Disease penetrance (proportion of mice developing a clinical score >1 for more than three consecutive days) was 100% in *Zfp36+/+* mice and 0% in *Zfp36aa/aa* mice. Cumulative disease severity, calculated as area under the curve (AUC) of the plot of disease score against time, was reduced by 70% in heterozygous *Zfp36+/aa* mice (AUC of 37.6±14.1, compared with 123.8±7.4 in Zfp36+/+ mice, p<0.01). This finding is consistent with the dominant anti-inflammatory function of the constitutively active form of TTP,^15^ and suggests that a moderate alteration in the equilibrium between phosphorylated and unphosphorylated TTP has anti-inflammatory consequences.

### Roles of stromal and haematopoietic TTP in protection against experimental arthritis

**Figure 5:**
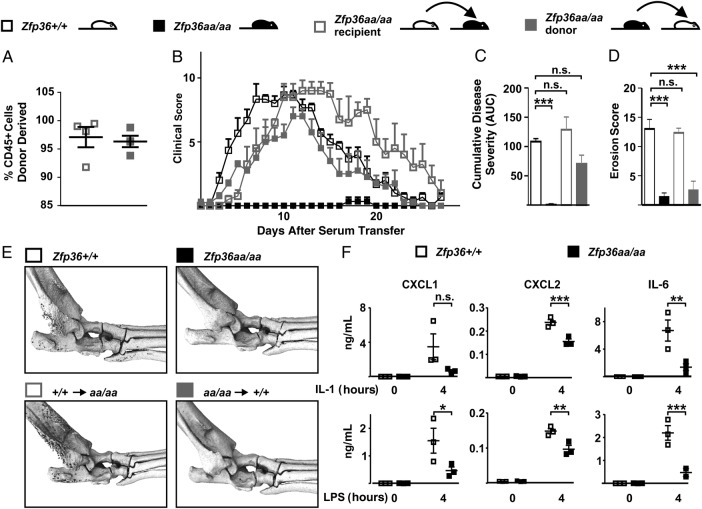
Enhanced tristetraprolin (TTP) function in both haematopoietic and non-haematopoietic compartments is required for protection against experimental arthritis. Bone marrow chimaeras were created between *Zfp36+/+* and *Zfp36aa/aa* mice. (A) 8 weeks after reconstitution of lethally irradiated recipient mice, chimerism of haematopoietic cells was assessed by flow cytometry of CD45.1 and CD45.2 markers. (B) Arthritis was induced as in figure 3, and clinical scores were monitored over 26 days. (C) Cumulative disease severity was calculated for each individual mouse as area under curve (AUC) of the plot of clinical score against time. Graph represents mean±SEM (n=3 for *Zfp36+/+* and *Zfp36aa/aa*, n=4 for chimaeras. n.s., not statistically significant; ***p<0.005. (D) Bone erosion was quantified in *Zfp36+/+*, *Zfp36aa/aa* and chimeric mice. (E) Representative μCT images of mice at d26. (F) Joints of healthy *Zfp36+/+* and *Zfp36aa/aa* mice were digested and adherent cells passaged three times, after which fewer than 2% of cells were CD45+. Cells were then left untreated or stimulated with 10 ng/mL interleukin (IL)-1β (upper) or LPS (lower) for 4 hours. Secreted CXCL1, CXCL2 and IL-6 were measured. Graphs represent mean±SEM (n=3). *p<0.05; **p<0.01; ***p<0.005 (Mann-Whitney U test).

To investigate the functions of TTP in haematopoietic and non-haematopoietic compartments, bone marrow chimaeras were generated between *Zfp36+/+* and *Zfp36aa/aa* mice, and STIA experiments performed. On average, 97% of CD45+ cells in both chimaeras were donor-derived (figure 5A). In *Zfp36+/+* mice, STIA developed and resolved over 26 days, while there was no evidence of disease in *Zfp36aa/aa* mice over the same interval (figure 5B). Despite subtle differences in the timings of disease onset and resolution (figure 5B), neither mice expressing TTP-aa in haematopoietic cells (filled grey symbols) nor in non-haematopoietic cells (open grey symbols) were protected from STIA, at least at the level of macroscopic symptoms (figure 5C). However, the presence of TTP-aa in haematopoietic cells conferred protection against bone erosion (figure 5D, right panels of figure 5E).

The lack of protection from STIA in mice expressing TTP-aa only in the haematopoietic compartment implied that TTP may function in non-haematopoietic cells to inhibit inflammation. Joint stromal cells were isolated from Zfp36+/+ and Zfp36aa/aa mice and challenged with either IL-1β or lipopolysaccharide (LPS; figure 5F). The expression of IL-6, CXCL1 and CXCL2 was significantly decreased in *Zfp36aa/aa* stromal cells stimulated with either IL-1β or LPS, except in the case of IL-1β-stimulated CXCL1, where the difference of expression did not achieve statistical significance (p=0.06).

### Therapeutic targeting of TTP phosphorylation

To investigate whether pharmacological disturbance of the equilibrium between phosphorylation and dephosphorylation of TTP conferred protection against STIA, we used two chemically distinct compounds reported to activate PP2A by disrupting its interactions with inhibitory protein complexes.^34^ ^35^ COG1410, an apolipoprotein E peptide mimetic, exerts protective effects in experimental models of neuroinflammation.^36^ ^37^ AAL(s) is a lipid derivative of the immunosuppressant FTY720 (fingolimod). Unlike its parent compound, it does not influence sphingosine 1 phosphate signalling and has no effects on lymphocyte trafficking. Instead, its anti-inflammatory effects have been attributed to activation of PP2A and subsequently TTP.^35^ ^38^ ^39^

**Figure 6:**
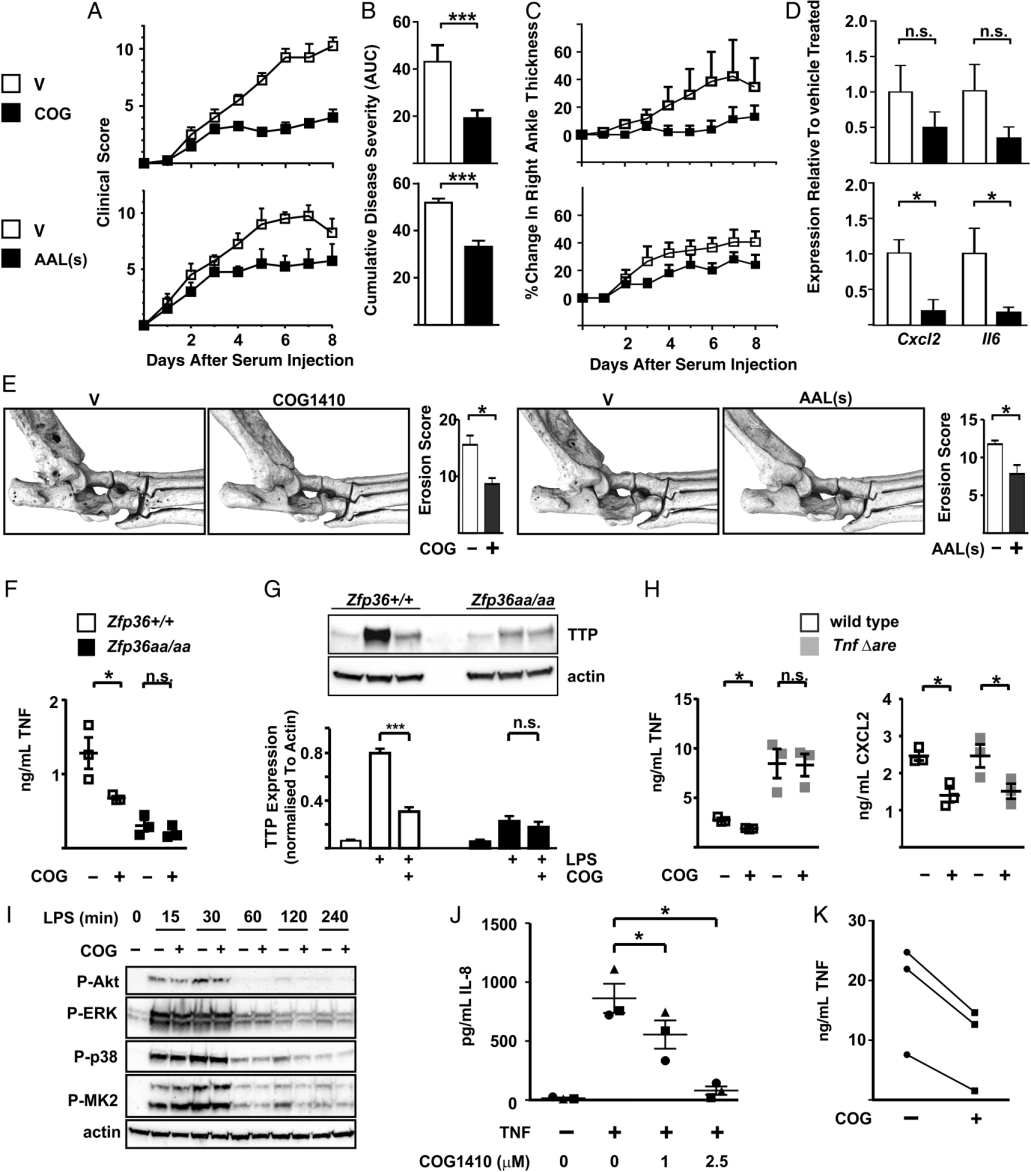
Therapeutic targeting of tristetraprolin (TTP) phosphorylation. Arthritis was induced in wild-type mice by injection of K/BxN serum as in figure 3. From d2 mice were treated daily with vehicle or COG1410 (upper panels), vehicle or AAL(s) (lower panels). Control mice were injected with vehicle (dimethyl sulfoxide in the case of AAL(s), phosphate-buffered saline in the case of COG1410. (A) Clinical score was monitored over 8 days. (B) Cumulative disease severity was calculated as in figure 5. (C) Right ankle thickness was measured. (D) At the end of the experiment, RNA was isolated from wrist joints, *Cxcl2* and *Il6* mRNAs were measured by quantitative PCR with normalisation first against *Gapdh* then against vehicle-treated control. Graphs represent means±SEM of four mice in each treatment group. n.s., not significant; *p<0.05; ***p<0.005 (Mann-Whitney U test). (E) Representative μCT images of ankles of mice treated to day 8 with vehicle, COG1410 or AAL(s). Bone erosion was scored in blinded manner by three independent observers. Graphs show means±SEM. *p<0.05 (Mann-Whitney U test). (F) *Zfp36+/+* and *Zfp36aa/aa* BMMs were treated with LPS for 8 hours with or without 10 μM COG1410 (COG). Secreted tumour necrosis factor (TNF) was measured by ELISA. Graph represents mean±SEM from three independent BMM isolates of each genotype. n.s., not significant; *p<0.05 (Mann-Whitney U test). (G) Whole cell lysates were prepared from *Zfp36+/+* and *Zfp36aa/aa* BMMs treated as indicated, and TTP detected by western blotting. Protein abundance was estimated by scanning densitometry, with normalisation against the loading control actin. The graph represents mean±SEM from three independent experiments. n.s., not significant; ***p<0.005 (Mann-Whitney U test). (H) Litter-mate control and TnfΔARE BMMs were treated with LPS for 8 hours with or without 10 μM COG1410 (COG). Secreted TNF and CXCL2 were measured by ELISA. Graphs represents mean±SEM from three independent BMM isolates of each genotype. n.s., not significant; *p<0.05 (Mann-Whitney U test). (I) *Zfp36+/+* BMMs were treated with LPS for 0–4 hours with or without 10 μM COG1410 (COG). Whole cell lysates were western blotted for phosphorylated forms of Akt, ERK, mitogen-activated protein kinase (MAPK) p38 and MK2, and for actin as a loading control. Representative of two independent experiments. (J) Rheumatoid arthritis (RA) synovial fibroblasts were stimulated for 8 hours with 1 ng/mL TNF in the presence of 0, 1 or 2.5 μg/mL COG1410. Secreted IL-8 was measured by ELISA. Circles, squares and triangles represent independent RA synovial fibroblast cultures derived from three different patients. Each individual symbol represents the mean of triplicate measurements. n.s., not significant; *p<0.05 (two-way analysis of variance). (K) Monocyte-derived macrophages were isolated from three volunteers and stimulated with LPS for 8 hours with or without 10 μM COG1410 (COG). Secreted TNF was measured by ELISA.

C57BL/6 mice were treated with vehicle, COG1410 or AAL(s) from day 2 after the injection of K/BxN serum. Both COG1410 and AAL(s) reduced disease progression (figure 6A), cumulative disease severity (figure 6B) and ankle swelling (figure 6C). AAL(s) significantly decreased the expression of the pro-inflammatory genes *Il6* and *Cxcl2* in joints (figure 6D). This effect did not reach statistical significance in COG1410-treated mice. Βoth PP2A agonists protected against bone erosion (figure 6E). A control scrambled peptide with the same amino acid composition as COG1410 had no effect on disease progression (see online supplementary figure S3).

In vitro experiments investigating the mechanisms of response to PP2A agonism employed COG1410, which had stronger anti-inflammatory effects in vivo. COG1410 significantly decreased expression of TNF by *Zfp36+/+* bone marrow-derived macrophages (BMMs), whereas in *Zfp36aa/aa* BMMs the expression of TNF was comparatively low, and was not further decreased by COG1410 (figure 6F). COG1410 decreased the expression of wild-type TTP, but did not affect the weak expression of TTP-aa (figure 6G). These observations support the hypothesis that activation of PP2A and dephosphorylation of serines 52 and 178 both activates and destabilises TTP. Effects of COG1410 were also tested in BMMs derived from *Tnf-Δare* mice, in which the TTP binding site is specifically deleted from *Tnf* mRNA.^12^ Elevated expression of TNF in *Tnf-Δare* BMMs was insensitive to COG1410, whereas the expression of CXCL2 remained sensitive to COG1410 (figure 6H). COG1410 did not influence Akt, extracellular signal-regulated kinase (ERK), MAPK p38 or MK2 phosphorylation in response to LPS (figure 6I). Therefore, in vitro anti-inflammatory effects of COG1410 require an intact TTP binding site within the target mRNA, depend on the ability to modulate TTP phosphorylation state, but do not involve impairment of signalling events upstream of TTP phophorylation. In three independently derived RA synovial fibroblast lines, COG1410 dose-dependently inhibited TNF-induced IL-8 expression (figure 6J). In human monocyte-derived macrophages, COG1410 also decreased the LPS-induced expression of TNF (figure 6K), suggesting that promotion of the dephosphorylation and activation of TTP may also exert anti-inflammatory effects in these cells.

## Discussion

Here and elsewhere,^30^ ^40^ the anti-inflammatory effector protein TTP has been shown to be strongly expressed at sites of active inflammation, including RA synovial lining cells. This prompted the question of “Why TTP is not acting to reduce TNFα expression in rheumatoid synovial macrophages”.^30^ A likely answer is that aberrant activation of MAPK p38 in the RA synovium leads to the accumulation of TTP in a phosphorylated and inactive form, so that it is incapable of downregulating TNFα and other targets. This conclusion is based on the observations that: (1) TTP expression was higher in inflamed than non-inflamed synovial tissue (figure 1A, B); (2) strong expression coincided with activation of the MAPK p38 pathway (figure 1F); (3) precise, genetically mediated blockade of MAPK p38-dependent phosphorylation of TTP powerfully protected mice against experimental arthritis (figures 3–5). The dramatic, inflammation-resistant phenotype of the genetically modified *Zfp36aa/aa* mouse in this robust experimental model is, to our knowledge, unprecedented for such a small genetic alteration (substitution of two codons).

Bone marrow chimaera experiments demonstrated that protection from experimental arthritis was dependent on enhanced function of TTP in both haematopoietic and non-haematopoietic compartments (figure 5). This finding is consistent with reports that global *Zfp36* gene disruption led to spontaneous inflammatory arthritis,^9^ whereas myeloid-specific *Zfp36* gene disruption did not.^10^ ^11^ It was recently shown that TTP negatively regulates expression of several inflammatory mediators in murine embryonic fibroblasts.^41^ Here, we show that murine joint fibroblasts containing the constitutively active form of TTP expressed lower amounts of IL-6, CXCL1 and CXCL2 in response to inflammatory stimuli. These attenuated responses of stromal cells may help to explain the delay in onset of arthritis in chimaeras expressing TTP-aa in their non-haematopoietic compartment. TTP protein was detected in a subset of synovial fibroblasts of patients with RA (figure 1). Therefore, it is likely that TTP functions as a suppressor of inflammation in at least some joint fibroblasts. The strong expression of TTP protein in vascular endothelial cells was also striking, and may point to a role in restraint of vascular activation, via downregulation of cytokines, chemokines and/or adhesion molecules.^40^ ^42^ ^43^

The bone marrow chimaera experiments also revealed uncoupling between joint inflammation and bone erosion (figure 4). When TTP-aa was present in haematopoietic cells, mice were not protected from macroscopic inflammation, but were strongly protected against bone erosion. Inflammatory osteolysis is dependent on MAPK p38.^44^ Osteoclast activation and bone erosion were increased in mice lacking the dual specificity phosphatase DUSP1, and therefore having dysregulated MAPK p38 activity.^45^ In vivo delivery of TTP using an adenoviral vector conferred protection against experimentally induced osteolysis.^44^ ^46^ It is therefore likely that the MAPK p38-TTP pathway controls the differentiation and/or activation of osteoclasts. The absence of a bone phenotype of *Zfp36aa/aa* mice under resting conditions suggests that such a role would likely be restricted to inflammatory contexts.

We suggest that promoting the dephosphorylation of TTP represents a novel strategy for treatment of chronic inflammatory pathologies such as RA. Potential advantages of this approach are: (1) that it may avoid some of the pitfalls of targeting upstream signalling events; (2) that it requires no assumptions about the factors that drive inflammation or the signalling pathways actually responsible for the phosphorylation and inactivation of TTP in the RA synovium; (3) that it capitalises on the accumulation of TTP at sites of inflammation,^30^ ^40^ a pool of dormant anti-inflammatory effector protein that is ripe for re-activation. This is in keeping with the philosophy of harnessing edogenous off-switches and anti-inflammatory mechanisms, rather than targeting the activators of inflammation.^47^ Proof of principle is provided by two chemically distinct compounds that are reported to activate PP2A, and which reduce inflammation and bone erosion in an experimental model of RA (figure 6A–E). PP2A is a pleiotropic enzyme complex with multiple substrate phospho-proteins and cellular functions,^48^ therefore, the mechanism of anti-inflammatory action of PP2A agonists is open to question. Our in vitro data support the concept that activation of PP2A exerts anti-inflammatory effects at least in part via the activation of TTP (figure 6F–I). Future studies will investigate relationships between TTP expression and phosphorylation state, disease activity and response to therapy. More also needs to be known about mechanisms of recognition and dephosphorylation of TTP by PP2A. The data reported here suggest that these will be fruitful areas to explore.
