# Supplementary material for: Treatment of inflammatory arthritis via targeting of tristetraprolin, a master regulator of pro-inflammatory gene expression
Source: Ann Rheum Dis. 2017 Feb 17;76(3):612–9. doi: 10.1136/annrheumdis-2016-209424 (PMC5446007; doi:10.1136/annrheumdis-2016-209424)

ROSS ET AL. SUPPLEMENTAL MATERIAL.

METHODS.

Human tissue. Synovial tissue biopsies were obtained from patients undergoing ultrasound-guided arthroscopy. Samples were designated "normal" if histological inspection revealed no macroscopic evidence of inflammation, and absence of inflammatory pathology was confirmed by clinical follow-up. All participants gave written informed consent, and the study was approved by the National Research Ethics Service Committee West Midlands - The Black Country.

Immunofluorescence. Human synovial tissue was frozen in OCT compound (Takara) and prepared as previously described [1]. Slides were stained for confocal microscopy by blocking in 10% horse serum (Sigma-Aldrich) in TBS for 1 hour at room temperature. Primary antibodies were added in blocking buffer and slides incubated overnight at 4°C (see Table 1). Slides were washed in TBS and stained with secondary antibodies (see Table 2) in blocking buffer containing 10 μg/ml Hoechst 33342 (Life Technologies). Slides were then washed in TBS and mounted with ProLong Diamond Antifade (Life Technologies). Images were obtained using a 10x or 40x objectives on an LSM 510 microscope (Zeiss), and processed using the Zeiss LSM Image Examiner software.

In vivo models. All mice were maintained and housed under conventional conditions in the Biomedical Services Unit at the University of Birmingham. Experimental protocols were performed under Home Office guidelines and project licence 40/8003. *Zfp36aa/aa* [2] and TNFΔARE [3] strains have been previously described. Single animals were considered as experimental units. Study group sizes were based on estimates of effect size from a pilot experiment, using three mice of each genotype.

For the air pouch acute inflammation model, mice were subjected to light anesthesia using halothane. A localized dorsal cavity was created by s.c. injectionof 3 ml of air. At d4, a further 1.5 ml of air was injected s.c. at the same site. At d7, 1 mg zymosan in 100 μl of PBS was injected into the air pouch. Mice were humanely culled after 1 h or 4 h. Pouches were injected with 1 ml PBS/EDTA, massaged, and exudates collected. Cells were counted by trypan blue staining and hemocytometry.

Inflammatory arthritis was induced by transfer of 75 μl K/BxN serum i.v. as previously reported [1]. Arthritis progression was assessed by weight change, observed clinical scoring of inflamed joints and measurement of joint swelling using callipers (POCO-2T, Kroeplin GmbH). Mice were given pain relief (60 μg/Kg Vetegesic) if they reached a certain combined clinical score (based on weight, grimace, joint count

and degree of inflammation) or showed signs of significant impairment in motility. At peak of inflammation (approximately day 10) or at resolution (day 28 or later) mice were humanely sacrificed.

Congenic whole BM chimeric animals (WT, CD45.1; *Zfp36aa/aa,* CD45.2) were generated as previously described [4]. Reconstituted animals were checked for chimerism at 8 weeks post transfer by flow cytometry of peripheral blood. Chimeras were challenged with athritogenic serum as described above.

In some experiments mice were given 5 daily injections of vehicle or PP2A activating compounds (10 mg/kg of AAL(s)or 5 mg/kg of COG1410) after the appearance of symptoms (usually two days after serum transfer). Control micewere injected with vehicle (DMSO in the case of AAL(s), PBS in the case of COG1410). 24h after the last treatment, mice were humanely sacrificed.

Joint digests. One hind leg was cleaned of all tissue and the upper and lower leg bones separated, whilst ensuring the surface of the knee joint remained intact. The exposed joint was digested in 1.5 ml of RPMI containing 2% FCS, 2.5 mg/ml collagenase D (Roche) and 20 µg/ml DNAase (Sigma-Aldrich) for 45 mins at 37°C with constant agitation. Digests were then filtered through 100 µm cell strainer and cells kept on ice. Bones were further digested for 30 min at 37°C in 1.5 ml RPMI containing 2% FCS, 2.5 mg/ml collagenase/dispase (Roche) and 20 µg/ml DNase (Sigma-Aldrich) with constant agitation. 50 µl 0.5M EDTA solution was added and incubated for a further 5 min, before filtering the digest through the same cell strainer as before, pooling the cells with those collected from the previous incubation. Combined cells were spun down, red cells lysed for 5 mins in ACK lysis buffer (Gibco) and cell counted and stained for flow cytometry. Mesenteric lymph nodes were collected from two donors, processed in the same way and cells used as single colour controls for flow cytometry. Infiltrating cells were assessed using F4/80-PE, CD3-PerCP-Cy5.5, CD11c-PE-Cy7, CD11b-eFluor450, GR1-APC and CD19-ApC-Cy7 (all eBioscience) and B220-PE-Dazzle510 (Biolegend). Macrophage populations in joint digests were identified as previously described [1].

In vitro assays. Bone marrow derived macrophages (BMM) were cultured as previously described [2]. Human macrophages were generated from peripheral blood monocytes as previously described [5]. Macrophages were stimulated with 10 ng/ml LPS (Alexis) in the presence or absence of PP2A activating compounds. Cell culture supernatants were collected, cells washed once in ice-cold TBS, and resuspended in lysis buffer for immunoblotting.

Histology. Whole mouse legs were fixed for 24 hours in 10% formalin solution (Sigma-Aldrich) and decalcified at room temperature in 10% EDTA (pH 7.4). Decalcification was confirmed by X-ray analysis,

legs were embedded in paraffin and 5 µm microtome sections cut. Sections were stained with haematoxylin and eosin for cellular histology or with safranin O and fast green to highlight cartilage. Histology was imaged using an Axio Scan Z1 (Zeiss) slide scanner.

To identify osteoclasts, tartrate-resistant alkaline phosphatase (TRAP) staining was performed. Slides were incubated for 2.5 hours at 37°C in TRAP staining solution (1 litre comprising 9.2 g anhydrous sodium acetate, 11.4 g L-(+) tartaric acid, in distilled water, 2.8 ml glacial acetic acid, pH to 4.7 with 5M sodium hydroxide) containing 1.5 ml naphthol AS-MX phosphate substrate mix (30 mg naphthol AS-MX phosphate in 1.5 ml ethylene glycol monoethyl ether) and 180 mg fast red violet salt.

Micro-CT. Hind limbs were imaged using a Skyscan 1172 micro-CT scanner (Bruker) using X-ray beam settings of 600kV source voltage, 167µA source current. Projections were taken every 0.45 degrees at 600 ms exposure, with an image pixel size of 13.59 µm. Image volumes were reconstructed using the Feldkamp algorithm (NRecon software, v1.6.1.5, Buker) having applied beam hardening correction. A radiodensity range of -300 to 3000 HU was chosen to isolate the bony structures from the imaging medium and CTAnalyser software v1.12 (Bruker) was used to extract an isosurface mesh representation of the reconstructed mirco-CT slices. MeshLab v1.3.2 (open source software developed with the support of the 3D-CoForm project) was used to modify the raw meshes and poisson surface reconstruction to generate a smooth, uniformly sampled surface mesh that preserved the original surface topography. The samples were shaded in MeshLab using ambient occlusion.

Micro-CT meshes were visualised in MeshLab and scored blinded by 3 independent researchers. Meshes were divided into 3 regions: Heel (compromising the calcaneus, centrale, distal tarsals, tibulae and astagalus but excluding the tibia and fibula), metatarsals and phalanges (excluding the claws). Each region was scored for erosion (0 = normal, 1 = roughness, 2 = pitting, 3= full thickness holes) and the extent of the area affected (0 = none, 1 = a few small areas, 2 = multiple small-medium areas, 3 = multiple medium-large areas). The 2 scores were then multiplied together for each region to give a maximum score per paw of 27. The average score and standard deviation from the 3 researchers is shown.

mRNA quantification. Wrist or ankle joints were snap frozen in liquid nitrogen at the time of dissection and stored at -80°C. Joints were pulverised to a fine powder in liquid nitrogen using a FreezerMill 6770 (Spex Sample Prep) and mRNA isolated using Trizol reagent (Life Technologies) as per manufacturer's instructions. cDNA synthesis and Real Time PCR was performed as previously described [2].

Analysis of Protein Expression. Secreted factors in cell supernatants were quantified using multiplex bead assay (Biorad) or ELISA (eBioscience and Peprotech) according to manufacturers' instructions. Cell lysates were resolved on SDS-PAGE gels, probed with primary antibodies, and immunoreactive proteins were visualized with HRP-coupled secondary antibodies and chemiluminescence reagents (Bio-Rad, Pierce or Cell Signaling Technologies). Blots were visualized using the ChemiDoc MP imaging system (Bio-Rad).

Statistics. Statistical analysis of data was performed using Prism 6.0 (GraphPad). Pairwise comparisons used Mann Whitney U test. Analysis of larger data sets used ANOVA with Bonferroni correction for multiple comparison. The following marks are used throughout: *, $p < 0.05$; **, $p < 0.01$; ***, $p < 0.005$.

Table 1. Primary antibodies used for immunofluorescence

Antibody	Isotype (mouse unless stated)	Clone	Final concentration / dilution	Company
CD68	IgG2b	Y1/82A	5 μg/ml	BD Pharmingen
CD31	IgG2b	VM64	1 μg/ml	Santa Cruz
TTP	IgG2a	1A2	1:100	AbCAM
PDPN	IgG1	D2-40	1:100	AbD Serotec
CD248	IgG1	B1.35.1	1:3	In house antibody
pMK2	Rabbit IgG	27B7	1:200	Cell Signaling Technology

Table 2. Secondary antibodies used for immunofluorescence

Antibody	Fluorophore	Final concentration / dilution	Company
Anti-mouse IgG1	AlexaFluor 488	4 μg/ml	Life Technologies
Anti-mouse IgG2a	AlexaFluor 546	4 μg/ml	Life Technologies
	AlexaFluor 647	4 μg/ml	Life Technologies
Anti-mouse IgG2b	TRITC	5 μg/ml	Southern Biotech
	AlexaFluor 488	4 μg/ml	Life Technologies
Anti-rabbit IgG	AlexaFluor 546	4 μg/ml	Life Technologies
	AlexaFluor 647	4 μg/ml	Life Technologies

REFERENCES

1. Misharin AV, Cuda CM, Saber R, *et al*. Nonclassical Ly6C(-) monocytes drive the development of inflammatory arthritis in mice. *Cell Rep* 2014;9:591-604.

2. Ross EA, Smallie T, Ding Q, *et al*. Dominant Suppression of Inflammation via Targeted Mutation of the mRNA Destabilizing Protein Tristetraprolin. *J Immunol* 2015;195:265-76.

3. Kontoyiannis D, Pasparakis M, Pizarro TT, *et al*. Impaired on/off regulation of TNF biosynthesis in mice lacking TNF AU-rich elements: implications for joint and gut-associated immunopathologies. *Immunity* 1999;10:387-98.

4. Ross EA, Flores-Langarica A, Bobat S, *et al*. Resolving Salmonella infection reveals dynamic and persisting changes in murine bone marrow progenitor cell phenotype and function. *Eur J Immunol* 2014;44:2318-30.

5. Smallie T, Ricchetti P, Horwood NJ, *et al*. IL-10 inhibits transcription elongation of the human TNF gene in primary macrophages. *J Exp Med* 2010;207:2081-8.

SUPPLEMENTAL FIGURE LEGENDS.

Figure S1. RA synovial biopsy sections were stained with isotype control antibodies as indicated, then with appropriate fluorophore-coupled secondary antibodies.

Figure S2. Synovial biopsies were stained with antibodies as described in the legend to Fig. 1. Single color images are shown here to make clearer the localization of individual proteins. The biopsy shown in **S2A** is from a non-inflamed joint; all other biopsies shown are from patients with confirmed RA. Composite images are the same as those presented in Fig. 1. **S2F** is an additional image not included in the main manuscript for reasons of space. This shows a glancing longitudinal section of a blood vessel, with extensive co-expression of TTP and CD31 (a vascular endothelial marker).

Figure S3. As in Fig. 6A, serum transfer arthritis was induced in C57/BL6 mice. From day 2 after serum injection (when symptoms of joint inflammation had begun to appear), mice were treated daily with vehicle (PBS), 5 mg/kg COG1410 or 5 mg/kg of a control peptide with identical amino acid content but scrambled sequence (SCRAM). Clinical score was monitored until day 8.

Supplemental Figure S1.

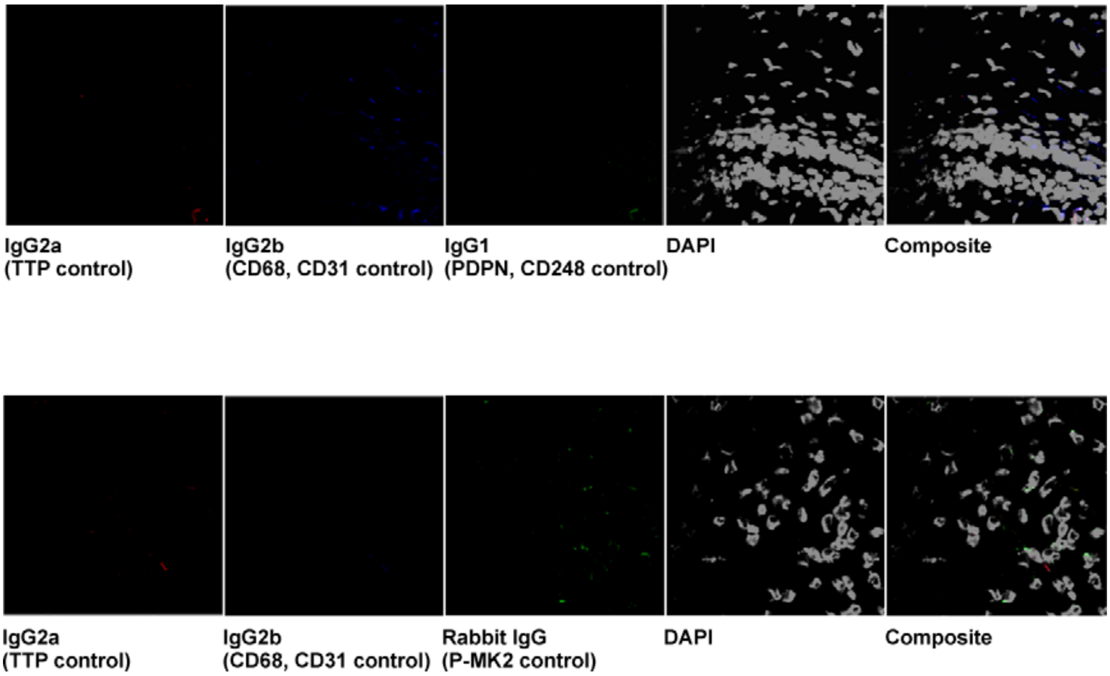

Supplemental Figure S2.

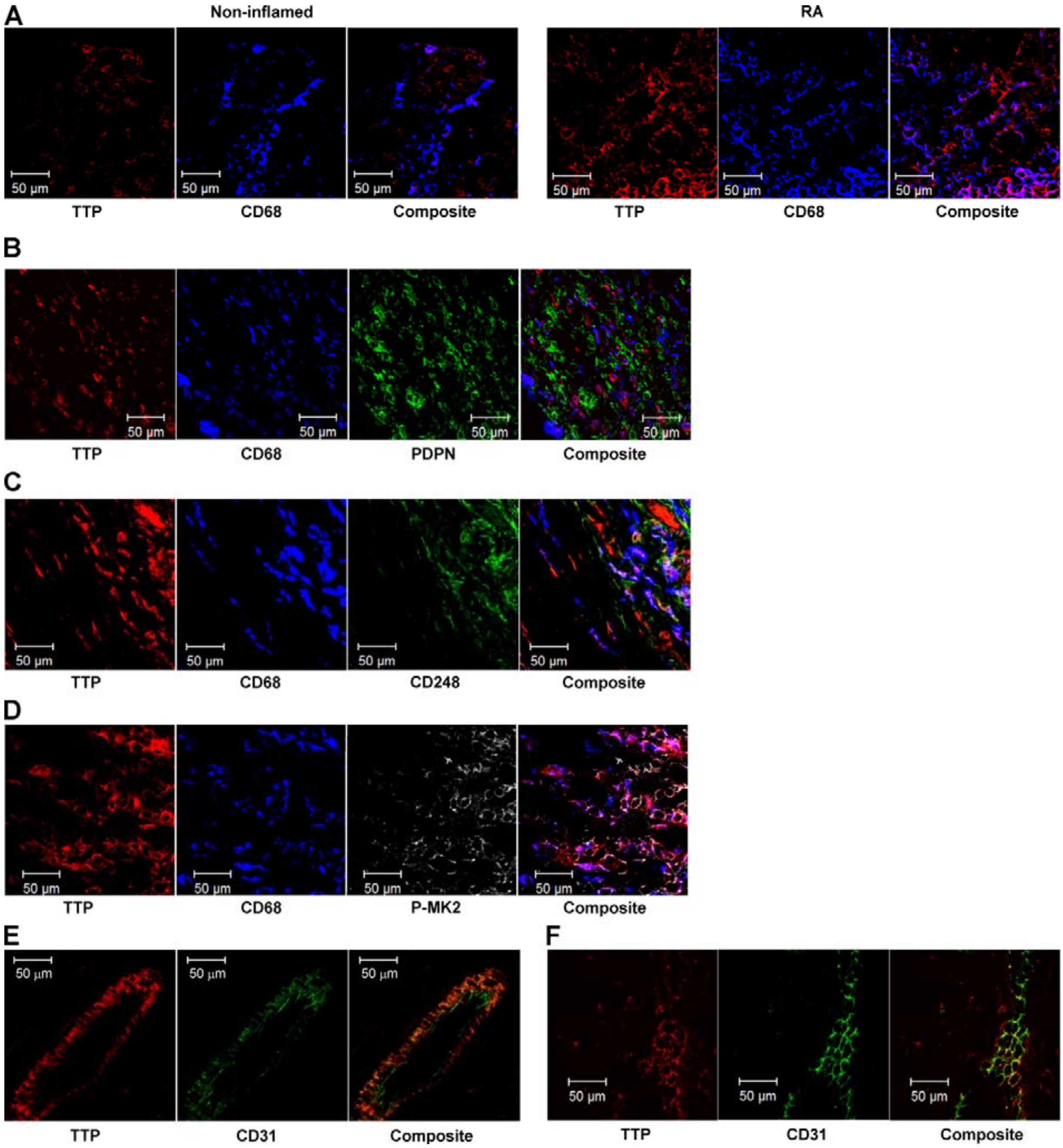

Supplemental Figure S3.

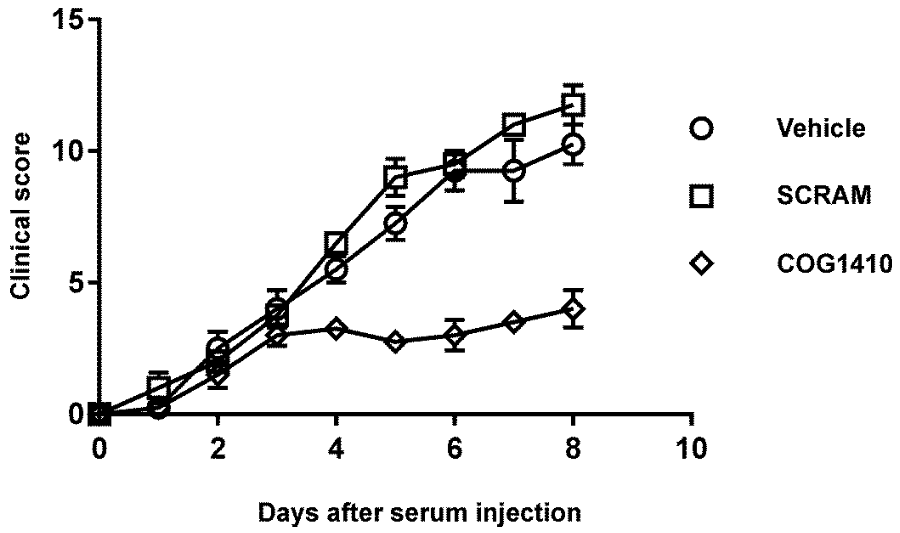

Supplement: Supplementary material [file annrheumdis-2016-209424supp001.pdf]
